# The Impacts of the High-Quality Workplace Relationships on Job Performance: A Perspective on Staff Nurses in Vietnam

**DOI:** 10.3390/bs8120109

**Published:** 2018-11-23

**Authors:** Khoa T. Tran, Phuong V. Nguyen, Thao T.U. Dang, Tran N.B. Ton

**Affiliations:** 1 Center for Higher Training and Research in Public Administration, International University-Vietnam National University, Ho Chi Minh City 700000, Vietnam; nvphuong@hcmiu.edu.vn; 2 School of Business, International University-Vietnam National University, Ho Chi Minh City 700000, Vietnam; dtuthao@hcmiu.edu.vn (T.T.U.D.); baotran09011996@gmail.com (T.N.B.T.)

**Keywords:** nurses, leader–member exchange, coworker relationships, high-quality workplace relationship, commitment, job stress, social impact, performance

## Abstract

Employees’ working relationships were long determined to be crucial to their overall wellbeing and performance ratings at work. However, a few studies were found to examine the effects of positive workplace relationships on employees’ working manners. This study aimed to investigate the effects of healthy workplace relationships on employees’ working behaviors, which in turn affect their performance. In doing so, an integrated model was developed to examine the primary performance drivers of nurses in Vietnamese hospitals and focus on the effects of high-quality workplace relationships on the working attitudes of the staff. This study analyzed a questionnaire survey of 303 hospital nurses using a structural equation modeling approach. The findings demonstrated the positive effects of high-quality workplace relationships on working manners including higher commitment, lower level of reported job stress, and increased perception of social impact. Notably, the results also demonstrated that relationships between leaders and their staff nurses make a significant contribution to the quality of workplace relationship and nurses’ performance. In addition, the social impact was illustrated to positively moderate the association between healthy workplace interactions and job stress; however, it had no significant effect on job commitment. Unfortunately, job commitment was surprisingly found to not be related to performance ratings. This paper provides some suggestions for the divergence of performance drivers in the hospital context in Vietnam.

## 1. Introduction

Managerial practices in the healthcare sector are facing considerable challenges to improve the performance of medical staff in the ever-growing hospital market conditions. In the opinion of Reference [1], nurses, among all healthcare practitioners, are the front-line care of patients in providing timely, quality health services in hospitals. Nurses also make up the largest human resource in healthcare organizations, and nursing performance remains a long-standing determinate of the quality of patient care. Therefore, the assessment of factors influencing the performance of nurses became essential for scholars and hospital management executives. This study, however, is particularly interested in Vietnamese hospital nurses.

Previous studies explored a variety of factors affecting job performance such as working environment, interpersonal relationships, job satisfaction, commitment, leadership styles, etc. [2,3,4]. This study focused on interpersonal relationships in the workplace and how these interactions affected the working manner and performance of employees. The extant scholarship indicates that employees who are well informed through relationships at work report higher levels of job satisfaction and commitment [5,6]. The higher the workplace interaction quality, the better informed employees are and, in turn, the less uncertain they are about tasks and goals and the better the perceived performance [7].

Of particular interest, the ways in which workplace relationships may influence the received information and resources were discussed through two primary sources of relationships with supervisors and colleagues. Prior studies showed evidence on the positive connections between these two relationship categories and the performance appraisal ratings of employees [8,9,10]. In light of this, employees can rely on their immediate leaders for an exchange of technical skills and resources, and the high-quality relationship with supervisors is associated with a higher level of trust, respect, obligation, support, and encouragement [11]. Otherwise, coworker relationships provide employees the source of emotional and instrumental support as they have an understanding of the internal working environment [12]. However, the literature on coworker relationships appears inconsistent regarding the healthcare sector, in which peer cohesion was less available between staff nurses who usually work on different rotating shifts [13,14]. Most of the related research focused on European countries and the United States.

Healthy workplace relationships receive a lot of interest since they are associated with the benefits of employees [15] and achieving the goals of the organization [16]. In addition, several previous studies also concentrated on examining the role of staff nurses’ participation in decision-making processes to improve the organizational process [17] and create a strong impact of health institution management models on clinical practice [18], as well as seeking clinical practice benchmarking to improve the quality of care [19], and identifying alternative models of effective funding systems in the national health service [20]. To further investigate this stream of research, Caillier [21] investigated the impact of high-quality workplace relationships on the overall performance of the organization through employee behaviors such as commitment, social impact, and job stress/exhaustion. However, the significance of healthy interpersonal relationships at work in Vietnam is of critical concern; however, it remains unexplored in research.

Since current research possesses little evidence about the expected impact of relationships at work on employee working manner, this paper aims to clarify the way in which healthy workplace interactions affect the working behaviors of staff nurses (commitment, stress level, the awareness level of social impact) and performance ratings by examining two subcategories of relationships individually. This study followed the same path as Li and Hung [22], who examined two subtypes of relationships separately and also grouped these types of relationships into one variable proposed by Hansen [23], allowing the consideration of separate effects and a whole effect.

## 2. Literature Review

### 2.1. High-Quality Workplace Relationships

Individuals are supposed to build and maintain positive relationships with others in the workplace [24]. The so-called workplace relationship is defined as the information exchange between individuals and groups who want to complete their goals [25] (p. 1379). Research long found a beneficial impact of the quality of employees’ workplace relationships with their supervisors and peers on organizations. The processing of information enhances the performance of an individual and the organization since well-informed employees are less uncertain about the target goals and make better decisions [7,26]. Certainly, some employees are better informed than others because the amount of information and resources received are likely affected to be by the quality of their relationships with their supervisors and coworkers. The current study examined two primary categories of the high-quality workplace relationship: supervisor–subordinate and peer relationships. Doing so allows the consideration of separate effects. In order to investigate an overall impact of the workplace relationship and to measure the extent to which each relationship subtype captures this construct. The study follows the same path as Reference [22] and also groups these relationships into one variable proposed by Reference [23].

### 2.2. The Leader–Member Exchange LME Theory

The leader–member exchange (LME) is the most widely accepted theory regarding supervisor–subordinate relationships [27]. It suggests that managers should encourage positive interactions between leaders and each of their employees through different exchange levels [28]. In general, these relationships vary with respect to quality, and high-quality workplace relationships rely on mutual trust, respect, obligation [29,30], and internal motivation [21] between organizational members. It follows that high-quality LME employees are likely to receive more support and attention from their leaders than those in low-quality LME due to more frequent communication. Sias [11] pointed out the positive association between LME and employee information experiences, providing some directions for examining how LME affects the quality of workplace relationships.

As a result, the quality of LME is linked to a variety of important individual and organizational outcomes. In the working environment where LME is of a high level, staff who are provided with technical knowledge and are encouraged with respect and feedback [31] are likely to exhibit a sense of motivation and commitment [32]. Allen [5] indicated that well-informed employees were more satisfied with their jobs, and the higher the level of satisfaction, the higher the degree of involvement [33] and lower intention to quit [5,34]. There is a variety of previous studies showing that a high-quality leader–member exchange maintains organizational commitment [35,36,37,38,39]. Azim and Islam [40] stated that perceived social support, which includes informational and instrumental support from others in networking, is an antecedent of nurses’ commitment in Saudi Arabia. However, private nurses exhibited a higher level of commitment than nurses working in the public sector. 

In addition, according to Reference [35], a high-quality or positive LME refers to a great exchange of resources and information between participants. Members who perceive a positive LME relationship deliver superior performances as they receive valued resources, opportunities, and support from their supervisors [41,42] to be more efficient and effective at work. More specifically, members of high-quality LME relationships tend to contribute more than what is required from their formal duties, and low-quality LME relationships make members “perform the more routine tasks” [28]. In line with this reasoning, high levels of LME are likely to provide employees with expectations about their ability in undertaking challenging task demands beyond their formal duties [43]. Hence, the following three hypotheses were proposed:

**Hypothesis** **1:** *The quality of the leader–member exchange has a significant and positive effect on high-quality workplace relationship.*

**Hypothesis** **2:** *The quality of the leader–member exchange has a significant and positive effect on employee commitment.*

**Hypothesis** **3:** *The quality of the leader–member exchange has a significant and positive effect on job performance.*

### 2.3. Coworker Relationships

The second major category of high-quality workplace relationships involves peer relationships, which are described as equivalent relationships [44] between similar status peers in the organization. These relationships provide significant attributes to a peaceful and productive work environment for several reasons. The coworker relationship (CWR) is referred to as a primary source of emotional support, career development [45], and instrumental support [11] as coworkers may have a clear understanding of the working experiences and conditions, as well as gossip about organizational information that cannot be obtained by external employees [46].

However, regarding the healthcare sector, staff nurses working in hospitals typically work long hours in rotating shifts with few breaks to ensure 24-hour patient care. Nurse working shifts may not follow traditional patterns of day and night, and nurses are required to work extra hours in specialized units such as surgery or intensive care [47]. In Vietnam, nurses are found to be involved in an “on-duty work schedule”, which means that staff nurses follow work hour regulations and ensures that 24 hours of continuous working is followed by a day off [48]. After all, staff nurses usually devote much of their free time to recover between shifts; hence, they have less time for interaction and resource exchange with their coworkers. Zedeck, Jackson, and Summers [49] indicated that night and rotating shift work, when compared to day work, was associated with adverse wellbeing outcomes, specifically, increased social isolation and lower levels of cohesion. In addition, Coffey, Skipper, and Jung [13] and Parkes [14] argued that peer cohesion was less available during rotating shifts between staff nurses. Of particular concern, Heath, Johanson, and Blake [50] demonstrated that the lack of effective communication, collaboration, and decision-making promotion adversely affected the quality of workplace relationships.

**Hypothesis** **4:** *Coworker relationships are proposed to be insignificantly related to high-quality workplace relationships.*

There are a large number of studies demonstrating the positive influence of peer interaction on job performance [22]. However, information and resource exchange through channels are found to be affected by different sectors. Regarding the healthcare industry, staff nurses usually work directly with their supervisors or doctors in their department [51] for technical information such as updated work plans, guidance, and job training. Moreover, staff nurses are in charge between different working shifts or are assigned to different tasks and care areas [52]. These are some reasons why a lack of interaction between staff nurses is likely to be observed when considering the healthcare sector. Hence, the lack of information exchange between peers happens to be insignificantly related to job performance.

**Hypothesis** **5:** *Coworker relationships are proposed to be insignificantly related to job performance.*

### 2.4. High-Quality Workplace Relationship, Social Impact, and Hospital Commitment

Job satisfaction and organizational commitment were obviously long found to be positively linked to the quality of workplace relationships including supervisor–subordinate relationships [5,53,54,55] and coworker relationships [56,57]. Specifically, Venkataramani et al., [58] argued that encouragement, support, and respect by managers within a high-quality workplace relationship organization facilitated the employee’s organizational attachment. Hackett and Guion [59] and Bass [35] proposed a positive interaction of coworkers on job satisfaction, which further increased the organizational commitment [60,61,62,63,64].

**Hypothesis** **6:** *The presence of high-quality relationships within the workplace will be associated with a higher level of organizational commitment among staff nurses*.

In addition, employees are considered to achieve an understanding about “the purpose of their work or what they believe is achieved in their work” through their interactions in the workplace [65]. Put differently, relationships at work make employees aware of the significance of the tasks they are performing. This line of reasoning is powered by the job design theories of “jobs as a collection of relationships, as well as a collection of tasks” [66] (p. 714). In this sense, a supportive working environment is proposed to contribute to the awareness of the differences that employees are making to others. Additionally, “the degree to which employees’ actions make a difference in the lives of other people, the extent to which employees’ efforts protect, promote or contribute to the welfare of others” is regarded as perceived social impact [67] (p. 51).

**Hypothesis** **7:** *Workplace relationships of high level are expected to be positively linked to a higher degree of realized social impact.*

According to Reference [68], in comparison with private employees, public employees are more likely to have a higher degree of spirit and motivation since they have a desire to serve, a so-called wish of creating benefits for others [69]. Apparently, not all public staff are perceived at the same level of social impact [67]. Specifically, regarding the healthcare sector, society usually considers nursing as a paramedical or an undervalued profession, whereby nurses simply follows doctors’ instructions. Public belief about nursing is partially created by the way the media portrays the healthcare sector, which includes only two groups of professionals: medical doctors and other health professionals serving doctors [70]. In the minds of most people, the role of staff nurses is restricted to medication giving, handling technology, and the measurement of vital signs [71]. As a result, staff nurses do not receive sufficient respect and recognition from society and, hence, may face stress, hardship, and bitterness when doing this profession. However, nurses still continue to stand firm in the workplace and live up to their own careers. This can be possibly regarded as occupational commitment, which is the tendency to remain working in the nursing sector and their loyalty to the nursing profession [72]. Explained by this kind of commitment, nurses endorse the value of nursing as they believe in their professional goals of doing good for society, making significant efforts in patient care, and having a sense of pride in their career [72,73], regardless of social valuation.

**Hypothesis** **8:** *Social impact is proposed to be not significant with commitment.*

### 2.5. High-Quality Workplace Relationships, Social Impact, and Job Stress/Exhaustion 

Individuals working in the healthcare industry are likely to face an extreme amount of stress including pain, morality, the inadequate number of nursing staff dealing with a large number of patients, work overload, decision-making based on insufficient information in emergencies, etc. [74]. Nguyen et al., [48] stated that nearly 50% of nurses experienced a high degree of stress in Vietnam. Additionally, the ratio of nurses suffering from exhaustion in Vietnam was found to be quite high in comparison with that of Japan and Finland [75]. Hence, job stress logically became the main factor in reducing professional efficiency and the quality of patient care. Blair and Littlewood [76] believed that the quality of workplace relationships was a potential contributor to employee stress. For example, employees working within high-quality workplace relationships are powered by instrumental resources and encouragement needed from supervisors and emotional support from peers that contribute to improving psychological empowerment [77]. Cohen and Wills [78] and Searle et al., [79] reported that interpersonal interactions at work reduced stress experiences among the staff. This is also similar to the study by Sveinsdottir et al., [80] where evidence was provided showing that lack of support from supervisors and peers, and less satisfaction with the head nurses generated stressful situations among nursing staff. More evidence of the positive connection between workplace relationships and job stress was recorded by Reference [81], who stated that the absence of support from the working environment scored higher for teaching hospitals and, thus, nurses in teaching hospitals reported a higher level of stress when compared to nurses in non-teaching hospitals.

**Hypothesis** **9:** *Nurses perceive that high-quality workplace relationships are negatively related to job stress/exhaustion levels*.

Since public staff perceive that they are doing good for others, they tend to contribute more to gain social impact [67], which in turn helps eliminate stress/exhaustion within the workplace environment. In addition, by creating a high-quality workplace relationship, managers who practice facets of transformational leadership ideally influence and inspire motivation, whereby individualized consideration, and intellectual stimulation are considered as a positive influential factor on employee performance [22,82]. It follows that meaningful tasks given by managers [83,84] help develop a positive attitude and stress relief [83]. The relationship between high-quality workplace relationship and job stress/exhaustion was found to be connected through social impact. Moreover, when employees are getting stressful/exhausted, they will not keep their commitment at workplace. Therefore, the following hypotheses are proposed:

**Hypothesis** **10:** *Staff nurses perceive that job stress and exhaustion are connected by social impact*.

**Hypothesis** **11:** *Staff nurses perceive that job stress and exhaustion have a negative impact on commitment.*

### 2.6. Job Performance and Commitment

Since employees who are more committed are less likely to be involved in withdrawal behaviors such as lateness, absenteeism [85], turnover intention, and are more willing to accept changes [86], it is evident that job performance would be potentially affected. Many studies paid attention to the relationship between commitment and job performance under different sample data. The majority of them pointed out the positive impact of employee commitment on performance, where committed individuals devote their time and energy to the pursuit of organizational goals and to delivering a superior performance [87]. Notably, Khan et al., [88] concluded that a higher employee commitment emerged as a predictor of better performance, considering the public sector of oil and gas in Pakistan. Other studies by Bakiev [89] and Mansour et al., [90] revealed the positive influence of organizational commitment on perceived performance among police officers in Krygyzistan and human resource management (HRM) practices in the Tunisian financial services industry, respectively. Especially regarding the public sector, Camilleri and van der Heijden [91] reported the same result. However, the findings of the positive relationship between commitment and job performance are inconsistent in the literature. For instance, the studies of Angle and Lawson [92], Steers [85], Mathieu and Zajac’s [93] and, more recently, the study by Biçer et al., [94] all observed a negative, insignificant, or even weak relationships between the two concepts. This variation can be explained by the inconsistency in measurements and constructs across the studies. Above all, consistent with the current literature in the nursing profession, nurse commitment is expected to be positively and significantly related to their performance in the Vietnamese context.

**Hypothesis** **12:** *Nurses perceive that commitment is positively connected to their job performance*.

**Figure 1:**
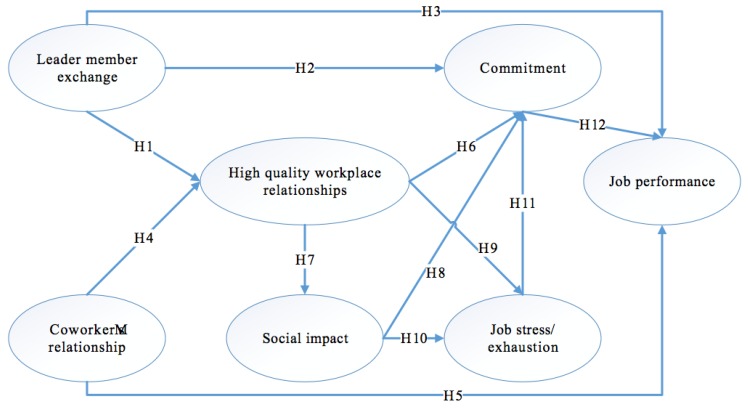
Research theoretical model.

Based on the analysis above, the research model was developed and is illustrated in Figure 1. It includes seven components. 

## 3. Methodology 

To test the research model and proposed hypotheses, this paper adopted a quantitative method where data were gathered by means of large-sample survey. The measures, sample and data collection, and data analysis technique are presented below.

### 3.1. Measures 

Referring to an extensive literature review, the study constructed a 33-item measurement scale to examine the latent variables observed. Firstly, the rate of the relationship with managers was assessed through the five-item scale from Graen and Uhl-Bien’s [31], which measures LME. Secondly, a three-item scale was adopted from Kram and Isabella [95] and typology relationships (three peer relationships distinguished by developmental and psychosocial function) [96,97] to assess coworker relationships. Each item was used to describe each type of coworker relationship by Sias [11]. Thirdly, high-quality workplace relationships were examined by the three-item scale from Hansen [23]. Fourthly, the social impact was assessed by the six-item scale following Bullock et al., [68] which included the definition of the social impact developed by Grant [67] (p. 51) (the actions of employees that make differences to others), and the same items built by Grant and Campbell [98]. Fifthly, instead of testing separately the two indicators of job stress/exhaustion by Steiber and Pichler [99], the study combined two indicators to make a five-item scale to measure job stress/exhaustion. This was in accordance with the theory of the Maslach Burnout Inventory developed by Maslach and Jackson [100]. Sixthly, a seven-item scale was applied by Bullock et al., [68] and Allen and Meyer [6] to examine the commitment. Finally, job performance was investigated through the four-item scale stated by Colquitt et al. [101]. A five-point Likert scale (1 = strongly disagree, 5 = strongly agree) was used to evaluate the level of respondents’ agreement.

### 3.2. Sample and Data Collection

In the sampling process, one of the most important questions is how many observations or respondents to use for the research sample. Deciding on the appropriate sample size is “an important and complex issue” which “depends on the statistical estimating precision needed by the researcher and the number of variables” [102]. Although larger sample sizes always provide better projection of the whole population, it is supported that a range from 200 to 400 is considered to be critical for multiple regression and path analysis [103]. Green [104] and Tabachmick and Fidell [105] suggest a rule of thumb for determining the sample size. Accordingly, sample size = 50 + (8 × number of measurement items). Because there were overall 33 items used for the measurement of the model’s constructs, a total of 314 observations was considered to be effective sample size. 

Since the purpose of the study was clarified, we contacted the Department of Health in both Ho Chi Minh City and Binh Duong province, Vietnam to get references for the list of hospitals. Then, we attempted to contact hospital managers to ask for help conducting the questionnaire survey. This enabled the delivery of the majority of the questionnaires to be done in person and to utilize the managers’ personal contact with many nurses. Consequently, researchers received the approval of hospital authorities from four hospitals in Ho Chi Minh City and two hospitals in Binh Duong province, Vietnam to conduct the questionnaire survey.

In order to assure ethical requirements, a cover letter was introduced in the first page of the questionnaire survey where the purpose of this study was clearly identified. It also involved the authors’ names, addresses, and university of the authors with the perspective of increasing the confidence of the staff nurse employees and for them to be familiar with whom they were answering as mentioned by Reference [106]. Researchers ensured that respondents’ information was kept confidential, used only for academic purposes. Firstly, the questionnaire was translated into Vietnamese and it was checked and corrected by an English lecturer. However, the first Vietnamese version was not easily understandable. Secondly, researchers conducted a pilot test of face-to-face interviews with 30 staff nurses in March 2018. The results of the pilot test enabled researchers to verify and modify the final questionnaire for easy understanding and improving the response rate under the Vietnamese context. 

With kind support from hospital managers, researchers distributed 80 questionnaires in each hospital. The managers assisted researchers in asking staff nurses to fill in the questionnaire. Researchers stopped by each hospital every weekend to receive the completed questionnaires. It took about three months to finish the data collection from April to June 2018. Out of the 480 questionnaires delivered to staff nurses in six hospitals, 382 responses were returned. However, 77 questionnaires were eliminated from the analysis because of missing data in the responses, effectively completing a response rate of 63.12% (303 responses) of usable responses for this study. A copy of the final questionnaire used in this paper is presented in Appendix A.

### 3.3. Data Analysis Technique

Firstly, exploratory factor analysis (EFA) was implemented to all scales together for a preliminarily assessment of dimensionality, convergence, and discriminant validity. Secondly, confirmatory factor analysis (CFA) was carried out to test the full measurement model which included seven constructs and their respective items.

## 4. Results

### 4.1. Demographic Characteristics 

Table 1 presents the demographic data of 303 respondents. Most of them were women (73%), and the majority of respondents were aged from 25 to 35 years old (71.6%). In addition, their experience in the nursing industry varied mostly from one to 10 years, with the rate at 63.7%. 

### 4.2. Reliability and Construct Validity

Firstly, Table 2 illustrates the descriptive statistics of data collection, including the mean and standard deviation. Specifically, in terms of the reliability, all the Cronbach’s alphas of the variables were greater than 0.7, and the corrected item-total correlations of all items were larger than 0.3, which confirms the reliability of the measurement requirements [107]. 

Secondly, exploratory factor analysis (EFA) was carried out to explore the possible underlying factor, and determine whether a set of variables consistently loaded on the same factor were based on strong correlations. Particularly, this test is used to “reduce the number of variables as the measurement indicators for the path analysis of overall model” [108]. According to Reference [109], factor loading can be classified as practical significance if it is greater than 0.5. Moreover, the Kaiser–Meyer–Olkin (KMO) should be higher than 0.5. Based on these criteria, the results of EFA led to dropping the following items: social impact 1 (SSI1), commitment 1 (COM1), LME4, and LME5. Therefore, the total number of items for testing the research hypotheses was 29 and the effective sample size needed to be at least 282 (50 + 8 × 29). In fact, 303 responses were appropriate and identified constructs of this research. After eliminating four items, the results showed that the constructs fully matched the design and each item was loaded mainly on its designate construct.

The KMO measure was 0.843, which was well within the acceptable limits. The Bartlett’s test of sphericity fulfilled the significance threshold (*p* < 0.000). Total extracted variance of 61.908% (>50%) indicated that seven factors were explained by 61.908% of the data variability. The remaining factor loadings of valid observed variables were above 0.5 and no major cross-loadings were reported. Then, the study applied Kaiser’s eigenvalue with a greater-than-one criterion to identify the seven factors that were extracted. 

Thirdly, an important step in CFA is the evaluation of the model fitness. Most of the model fit indices of CFA presented in Table 3 are in accordance with the acceptance requirement, except for a goodness-of-fit index (GFI) of 0.852, which was only considered as sometimes permissible, but was still within the range of threshold.

### 4.3. Convergent Validity and Discriminant Validity 

To examine the convergent validity of the measurement scale, two main indicators need to be considered: average variance extracted (AVE) and composite reliability (CR). According to Reference [112], the minimum value of AVE should be at least 0.5. However, if CR is greater than 0.6, the minimum value of AVE can be accepted as being at least 0.4, still assuring the construct convergent validity.

Table 4 illustrates that all estimates and AVEs of all factors were greater than 0.5, and CR values of all factors were good (> 0.7). This means that the convergent validities of all constructs were confirmed, and the reliability for all factors and items of the research model fully fulfilled the criteria.

In addition, Fornell and Larcker [112] suggest that the discriminant validity of the model is certified when the AVE of each measurement factor is higher than the square of the biggest correlation estimates of that factor with other ones (maximum shared variance, MSV). Table 4 also reports the satisfaction of all factors to the mentioned thresholds. 

### 4.4. Structural Equation Modeling and Hypothesis Testing 

The structural equation modeling (SEM) approach used the maximum-likelihood estimate to test the overall fit of the structural model. Applying the same requirement standards in CFA, the results in Table 3 state that most of the current indices of SEM lay well within the thresholds, and a GFI of only 0.842 is considered as sometimes permissible, but still acceptable. 

Before concluding on the fitness of the model, the *p*-values of correlations between factors were significantly checked. If the estimated standardized path coefficients summarized in Table 5 are statistically significant, the hypothesis will be accepted. At the 0.05 level of confidence, most of the path coefficients were positively and negatively significant, except for those of hypotheses H4, H5, H8, and H12. As observed from the results, LME had a positive and significant impact on high-quality workplace relationship (HWPR), COM, and job performance (JP). Thus, hypotheses H1, H2, and H3 were supported. The impacts of HWPR on COM and SSI were positively significant, while the impact of HWPR on job stress/exhaustion (JS) was negatively significant, indicating that hypotheses H6, H7, and H9 were all supported. Moreover, the results showed that SSI was significantly positively related to JS, supporting hypothesis H10. In contrast, there was no significant impact of coworker relationship (CWR) and COM on JP, SSI on COM, and HWPR on CWR. Hence, hypotheses H4, H5, H8, and H12 were all rejected.

In this study, it was found that the effect of LME on COM (path coefficient was 0.437) was more considerable than another predictor HWPR (path coefficient was 0.264). The path coefficient of SSI to JS (0.427) revealed its better contribution when compared to that of HWPR (−0.237).

## 5. Discussion

Among the two sources of workplace relationships, LME was indicated to be positively and significantly related to workplace relationship quality, employee commitment, and performance appraisal. These hypotheses support that high levels of LME, which feature mutual trust, emotional support, respect, and reciprocal influence create many positive outcomes including higher standard of patient care [77,113], greater job commitment, and stronger performance ratings [114]. These results are consistent with a majority of previous studies. Regarding nursing management, the findings agreed with Brunetto and Wharton [115] regarding positive connections between LME levels and the degree of nurses’ commitment and were in line with Han and Jekel [116] about the negative influence of high LME levels on nurse turnover rate in the United States. Additionally, in accordance with social exchange theory, the results support a recent study of Sepdiningtyas et al. [117] where LME was positively related to individual performance in the nursing profession. 

The results, however, pointed out the insignificant contribution of coworker relationships among Vietnamese hospital nurses in improving workplace relationship quality and job performance. This was explained by the fact that nursing has its own special characteristics when compared to other professions. The exchange of information and support is not highly valued among staff nurses since they usually follow their own work shifts or are in charge of different tasks and care areas. This line of reasoning is highly consistent with Blake [50], who argued that high levels of workplace environment were generated by effective communication and collaboration, and decision-making promotion among nurses. These reasons also support the insignificance of peer relationships and job performance. The study opposed Wong et al. [118] that weighted peer interactions, as the stronger driver on work engagement than supervisory relationships, were explained by the more frequent peer communications. The finding also contradicted previous studies in nursing by AbuAl-Rub [119] and Amarneh et al. [120] who reported a positive relationship between coworker social support and job performance among hospital nurses in the United States and Jordan, respectively. 

The results indicate that high-quality workplace relationship was negatively and significantly related to job stress, which is highly consistent with previous studies [16,77,78,79,80]. This is implied by the fact that, within positive workplace relationships, nurses are powered by instrumental resources and emotional support from supervisors and peers, which results in a lower level of reported stress. Moreover, the findings revealed that high-quality workplace relationships were considered as a significant driver of social impact. This is because a positive workplace relationship quality creates an understanding for the nurses of what they are doing and their awareness of the difference they are making in society. The results agree with Caillier [21] in the positive influence of high-quality workplace relationships on social impact among nurses. Additionally, there was a significant coefficient describing the positive interaction of social impact and job stress. A potential clarification could be that nurses who realize the impact they have on others put more effort and time toward preserving this aim. As a result, they reported a lower level of job stress or exhaustion. In the end, the study concluded on the mediating effect of social impact on the correlation between high-quality workplace relationship and job stress among staff nurses, which was highly consistent with Caillier [21]. Such workplace relationship quality stems from a transformational leadership style, which is described through the ideals of influence, inspiring motivation, individualized consideration, and intellectual stimulation [22,82,121]. Hence, transformational leaders provide staff with meaningful tasks and goals [83,84], which in turn enhances the social impact among nurses and contributes to relieving job stress [83].

Extracted from the results, it was observed that high-quality workplace relationships were positively and significantly related to nurse commitment. These findings support the previous findings ([122], [58] and [21]), they contended that interactions with leaders and peers in a positive workplace relationship enhanced employee psychological attachment to the organization. Furthermore, social impact was found as an insignificant influential factor on nurses’ attachment to the organization. The fact is that the nursing profession is likely to be undervalued by the public since they believe that nurses simply follow doctors’ instructions. However, nurses tend to believe in their professional goals to do good for society, contribute effortlessly in caring, and have a personal feeling of pride for their profession [72,73]. Consistent with this view, nurses remain working in the nursing sector and preserve the loyalty to their profession [72]. Since social impact is not a significant driver of nurse commitment, the result otherwise rejects [21] regarding the mediating effect of social impact on the relationship between high-quality workplace relationship and nurse commitment. 

Finally, the study demonstrated that organizational commitment emerged as an insignificant driver of the staff nurses’ performance in Vietnamese hospitals. This finding contradicts most of the previous research along this line. However, the study supported studies of Angle and Lawson [92], and Mathieu and Zajac’s [93], where the influence of commitment on perceived organizational performance was perceived negative, insignificant, or even weak. The variation can be explained by the way in which commitment and performance were conceptualized in each study. Gong et al. [123] examined different categories of organizational commitment and found out that, although affective commitment was explored to enhance job performance, continuance commitment was not. Meanwhile, Schrock et al. [124] reported the negative association between continuance commitment on job performance. Additionally, Biçer et al.,’s study [94] did not conclude a statistically significant interaction between affective commitment and perceived organizational performance. This is likely attributable to the fact that variable design shortcoming and other ambiguities suggest potential associations between kinds of commitment and job performance among Vietnamese hospital nurses. Several suggestions were proposed to enhance the understanding on this relationship, including investigating potential moderators [93], and examining different dimensions of performance [92] and commitment (affective, continuance, and normative).

## 6. Conclusions 

This paper aimed to examine the drivers of job performance among nurses in Vietnamese hospitals, paying attention to the significance of the quality of workplace interpersonal interactions. Since a positive workplace relationship is of crucial importance for the proper functioning and goal achievement of an organization, this study established an integrated model that aimed to explain the expected association of a high-quality workplace relationship, its subcategories, and working attitudes of Vietnamese hospital nurses. The findings showed that only the leader–member exchange relationship was a direct and significant predictor of high-quality workplace relationships and nurse performance, but not coworker relationships. Additionally, it also revealed that a healthy workplace relationship created a significant contribution to enhancing nurse commitment, relieving job stress/exhaustion, and increasing the perception of nurses about the social impact of the nursing profession. Unfortunately, the study did not demonstrate the significant relationship between commitment and job performance, and further research would need to be conducted to investigate the connections between the two concepts in the Vietnamese context. The divergence between the majority of previous studies on the commitment–performance relationship and that in the Vietnamese hospital context was also explained.

This study contributes to the extant research in two ways. Firstly, the paper firstly clarifies the process via which healthy workplace interactions affect the working behaviors of staff nurses (commitment, stress level, the awareness level of social impact) and performance ratings by examining two subcategories of relationships individually. Secondly, the study confirmed the significance of the association between workplace relationships, employee work attitudes, and job performance by considering different characteristics of the Vietnamese healthcare industry.

The findings of this study will provide valuable evidence and implications for healthcare management with regards to the importance of enhancing interpersonal interactions and performance ratings. Adequate attention should be paid to prioritizing solid interactions among staff nurses when they are demonstrated to have no contribution to the quality of workplace relationships and job performance among Vietnamese hospital nurses. Since such actions may improve the workplace relationship quality and job performance, this will in turn increase commitment, reduce stress levels, and improve nurses’ awareness of social impact.

In spite of the limitations, further studies may advance this line of research by widening the measurement variables of job commitment and performance to gain a deeper understanding about the connection between these two concepts among nurses in Vietnam. In addition, considering the generalizability of the findings, future research should examine other potential job performance drivers and any positive outcomes of coworker relationships in nurse performance and nurses’ working attitudes among various hospital sizes and characteristics. Another obvious limitation is that a larger-scale study across several countries should be conducted to eliminate the possible effects of the disparity between countries on the quality of healthcare services.

## Figures and Tables

**Table 1:** Demographic characteristics.

Characteristics	Number (*N* = 303)	Percentage
Gender		
Male	83	27.0%
Female	220	73.0%
Age		
Under 25	17	5.6%
From 25 to 35	217	71.6%
From 36 to 45	51	16.8%
Over 45	18	5.9%
Working experience		
Under 1 year	52	17.2%
From 1 to 10 year	193	63.7%
From 10 to 20 year	40	13.2%
From 21 to 30 year	18	5.9%

**Table 2:** Data description and reliability analysis

Variables	Numbers of items	Cronback’s alpha	Mean	Standard deviation
High-quality workplace relationship (HWPR)	3	0.818	3.69–3.92	0.817–0.912
Social impact (SSI)	6	0.860	4.02–4.25	0.762–0.904
Job stress/exhaustion (JS)	5	0.912	3.21–3.42	0.961–1.165
Commitment (COM)	7	0.860	3.28–4.16	0.600–0.978
Job performance (JP)	4	0.839	3.88–4.02	0.579–0.661
Leader–member exchange (LME)	5	0.814	3.52–3.66	0.808–1.014
Coworker relationship (CWR)	3	0.770	2.82–3.06	0.968–1.039

**Table 3:** Model fit indices in confirmatory factor analysis (CFA) and structural equation modeling (SEM).

Model fit indices	Thresholds	CFA	SEM	References
CMIN/DF	<3	2.109	2.238	Byrne [110]
RMSEA	<0.08	0.061	0.064	Bentler and Bonett [111]
GFI	≥0.90	0.852	0.842	Tabachnick et al. [105]
TLI	≥0.90	0.903	0.891	Bentler and Bonett [111]
CFI	≥0.90	0.915	0.904	Bentler and Bonett [111]

**Notes:** Chi-square/df (CMIM/DF); root-mean-squared error of approximation (RMSEA); goodness-of-fit index (GFI); Tucker–Lewis index (TLI); comparative fit index (CFI).

**Table 4:** Convergent and discriminant validity.

	CR	AVE	MSV	CWR	JS	COM	SSI	JP	HWPR	LME
CWR	0.789	0.563	0.058	**0.751**						
JS	0.914	0.680	0.045	0.078	**0.825**					
COM	0.869	0.528	0.288	0.177	−0.101	**0.727**				
SSI	0.874	0.584	0.236	−0.030	0.212	0.171	**0.764**			
JP	0.843	0.574	0.236	0.063	0.206	0.259	0.486	**0.757**		
HWPR	0.825	0.614	0.162	0.034	−0.053	0.347	0.315	0.403	**0.783**	
LME	0.852	0.661	0.288	0.240	0.044	0.537	0.284	0.469	0.330	**0.813**

Notes: composite reliability (CR); average variance extracted (AVE); maximum shared variance (MSV); average shared variance (ASV). The square root of AVE is shown in bold on the diagonal.

**Table 5:** Results of the model in SEM. Hypotheses 1–10 are denoted by H1–10.

Hypothesis	Path	Path coefficient	*p*-Value	Results
H1	HWPR ← LME	0.264	***	Supported
H2	COM ← LME	0.437	***	Supported
H3	JP ← LME	0.273	***	Supported
H4	HWPR ← CWR	−0.045	0.392	Rejected
H5	JP ← CWR	0.018	0.670	Rejected
H6	COM ← HWPR	0.219	**	Supported
H7	SSI ← HWPR	0.347	***	Supported
H8	COM ← SSI	0.012	0.867	Rejected
H9	JS ← HWPR	−0.237	**	Supported
H10	JS ← SSI	0.427	***	Supported
H11	COM ← JS	−0.084	**	Supported
H12	JP ←COM	0.013	0.795	Rejected

Note: * *p*-value < 0.10; ** *p*-value < 0.05l *** *p*-value < 0.001.

## Appendix A

### Research Questionnaire

#### Part 1: Respondent Demographic Information

**Table A1:** Demographic survey

Characteristics (N = 303)	
Gender	
Male	☐
Female	☐
Age	
Under 25	☐
From 25 to 35	☐
From 36 to 45	☐
Over 45	☐
Working experience	☐
Under 1 year	☐
From 1 to 10 years	☐
From 10 to 20 years	☐
From 21 to 30 years	☐

#### Part 2: Factors

In this part of the questionnaire, you are please required to indicate how much you agree or disagree with each of the following statements by circling the appropriate number that best reflects your answer. The scale is between 1 to 5, where: 1 = Strongly Disagree; 2 = Disagree; 3 = Neutral; 4 = Agree; and 5 = Strongly agree.

Please circle the option that best describes the level at which you agree or disagree with the following statements.

**Table A2:** Survey questionnaires.

**1. High Quality Workplace Relationship**						
		Strongly Disagree	Disagree	Neutral	Agree	Strongly Agree
HWPR1	In general, how would you describe relations at your workplace between nurse managers and nurses?	1	2	3	4	5
HWPR2	In general, how would you describe relationship at your workplace between workmates/ colleagues?	1	2	3	4	5
HWPR3	In general, how would you describe relationship at your workplace between nurses and doctors?	1	2	3	4	5
**2. Social Impact**						
		Strongly Disagree	Disagree	Neutral	Agree	Strongly Agree
SSI1	In my job I can help other people	1	2	3	4	5
SSI2	My job is useful to social	1	2	3	4	5
SSI3	The degree to which staff nurses’ actions make a different in the lives of other people.	1	2	3	4	5
SSI4	My work really makes other’s lives better’s	1	2	3	4	5
SSI5	I have positive impact on the others in my work on a regular basis	1	2	3	4	5
SSI6	My work has positive impact on a large number of people	1	2	3	4	5
**3. Job Stress/Exhaustion**						
		Strongly Disagree	Disagree	Neutral	Agree	Strongly Agree
JS1	How often do you come home from work exhausted?	1	2	3	4	5
JS2	How often do you find your work stressful?	1	2	3	4	5
JS3	I feel used up at the end of the workday	1	2	3	4	5
JS4	I feel emotionally drained from my work	1	2	3	4	5
JS5	Working with people directly puts to much stress on me	1	2	3	4	5
**4. Commitment**											
		Strongly Disagree	Disagree	Neutral	Agree	Strongly Agree
COM1	Willing to work harder to help hospital succeed	1	2	3	4	5
COM2	Proud to be working for this hospital	1	2	3	4	5
COM3	I would turn down another job to stay	1	2	3	4	5
COM4	Acceptance of hospital values, willingness to exert effort and desire to maintain membership in the organization	1	2	3	4	5
COM5	I would be happy to spend the rest of my career with this hospital	1	2	3	4	5
COM6	I enjoy discussing my hospital with people outsite of it.	1	2	3	4	5
COM7	I really feel as if this hospital’s problem are my owns	1	2	3	4	5
**5. Job Performance**						
		Strongly Disagree	Disagree	Neutral	Agree	Strongly Agree
JP1	How do you rate yourself in term of your ability to reach your goals	1	2	3	4	5
JP2	How do you rate yourself in terms of your performance potential among coworkers.	1	2	3	4	5
JP3	How do you rate yourself in terms of quality in your performance in regard to customer relationship?	1	2	3	4	5
JP4	How would you rate yourself in terms of quality of work you achieve?	1	2	3	4	5
**6. Leader Member Exchange**						
		Strongly Disagree	Disagree	Neutral	Agree	Strongly Agree
LME1	I usually know where I stand with my manager	1	2	3	4	5
LME2	My working relationship with my manager is effective	1	2	3	4	5
LME3	My manager recognizes my potential	1	2	3	4	5
LME4	I respect my supervisor’s knowledge and competence on the job	1	2	3	4	5
LME5	I like my supervisor very much as a person	1	2	3	4	5
**7. Coworker’s Relationship**											
		Strongly Disagree	Disagree	Neutral	Agree	Strongly Agree
CWR1	We primarily share the information necessary for us to get our jobs done. We provide very limited emotional or psychological support for each other.	1	2	3	4	5
CWR2	We share information about careers and provide feedback to each other, as well as needed job information. We help each other out as appears needed. We discuss personal topics such as family	1	2	3	4	5
CWR3	We rarely keep secrets from each other. We make a concerted effort to provide emotional support to each other. We talk frankly about nearly all topics. We let each other know how we feel about things at work and away from work	1	2	3	4	5
